# The Impact of Olive Oil Compounds on the Metabolic Reprogramming of Cutaneous Melanoma Cell Models

**DOI:** 10.3390/molecules26020289

**Published:** 2021-01-08

**Authors:** Cheila Brito, Ana Tomás, Sandra Silva, Maria Rosário Bronze, Ana Teresa Serra, Marta Pojo

**Affiliations:** 1 Unidade de Investigação em Patobiologia Molecular (UIPM) do Instituto Português de Oncologia de Lisboa Francisco Gentil E.P.E., 1099-023 Lisboa, Portugal; cheila__brito@hotmail.com (C.B.); acs.tomas@campus.fct.unl.pt (A.T.); 2 iBET, Instituto de Biologia Experimental e Tecnológica, 2780-157 Oeiras, Portugal; ssilva@ibet.pt (S.S.); mbronze@ibet.pt (M.R.B.); tserra@ibet.pt (A.T.S.); 3 Instituto de Tecnologia Química e Biológica António Xavier, Universidade Nova de Lisboa (ITQB NOVA), 2780-157 Oeiras, Portugal; 4 iMED, Faculdade de Farmácia da Universidade de Lisboa, 1649-003 Lisboa, Portugal

**Keywords:** cutaneous melanoma, oleic acid, homovanillyl alcohol, hydroxytyrosol, cell metabolism, molecular mechanisms

## Abstract

Cutaneous melanoma is the deadliest type of skin cancer, characterized by a high molecular and metabolic heterogeneity which contributes to therapy resistance. Despite advances in treatment, more efficient therapies are needed. Olive oil compounds have been described as having anti-cancer properties. Here, we clarified the cytotoxic potential of oleic acid, homovanillyl alcohol, and hydroxytyrosol on melanoma cells. Metabolic viability was determined 48 h post treatment of A375 and MNT1 cells. Metabolic gene expression was assessed by qRT-PCR and Mitogen-Activated Protein Kinase (MAPK) activation by Western blot. Hydroxytyrosol treatment (100 and 200 µM) significantly reduced A375 cell viability (*p* = 0.0249; *p* < 0.0001) which, based on the expression analysis performed, is more compatible with a predominant glycolytic profile and c-Jun N-terminal kinase (JNK) activation. By contrast, hydroxytyrosol had no effect on MNT1 cell viability, which demonstrates an enhanced oxidative metabolism and extracellular signal-regulated kinase (ERK) activation. This compound triggered cell detoxification and the use of alternative energy sources in A375 cells, inhibiting JNK and ERK pathways. Despite oleic acid and homovanillyl alcohol demonstrating no effect on melanoma cell viability, they influenced the MNT1 glycolytic rate and A375 detoxification mechanisms, respectively. Both compounds suppressed ERK activation in MNT1 cells. The distinct cell responses to olive oil compounds depend on the metabolic and molecular mechanisms preferentially activated. Hydroxytyrosol may have a cytotoxic potential in melanoma cells with predominant glycolytic metabolism and JNK activation.

## 1. Introduction

Cutaneous melanoma (CM) arises from the malignant transformation of melanocytes found in the skin [1]. Despite representing only 5% of all skin malignancies, CM is the most lethal type of skin cancer, accounting for more than 70% of all skin cancer-related deaths [2,3]. Indeed, the high mortality rate of CM patients is mainly related to its elevated potential to metastasize to surrounding tissues [4]. Melanoma progression and metastasis are influenced by the molecular and metabolic heterogeneity of melanoma cells, crucial in survival and acquisition of different nutrient sources [5]. In addition, this heterogeneity is also thought to play an active role in the development of therapy resistance, allowing melanoma cells to change and respond to microenvironmental cues [5]. In fact, it represents one of the main barriers to the efficiency of therapeutic approaches [6]. Currently, the available treatments for advanced-stage CM patients are surgical excision, when it is possible, targeted therapies, and immunotherapies. In the case of V-raf murine sarcoma viral oncogene homolog B1 (BRAF)-mutant melanomas, highly selective BRAF and mitogen-activated protein kinase (MEK) inhibitors have shown to improve patient survival [7,8,9]. However, only half of these patients demonstrate a positive response to targeted therapies, which tends to be limited over time [10]. On the other hand, immunotherapies targeting cytotoxic T-lymphocyte-associated antigen 4 (CTLA-4) and programmed cell-death protein 1 (PD-1) can lead to more durable responses compared to targeted therapies, also providing a survival benefit [9,11,12]. Nonetheless, response to immunotherapies is still limited, and acquired resistance is a problem for 40–70% of metastatic melanoma patients [11,13,14]. Thus, the identification of new potential therapeutic approaches is needed.

During recent years, several studies have described the medicinal properties of both olives and olive oil (reviewed in [15,16]). It is now widely accepted that monounsaturated fatty acids, phenolic compounds, and other phytochemicals present in olives and olive oil have numerous beneficial and protective effects, in regards to oxidative damage, inflammation, cardiovascular and neurodegenerative diseases, and cancer [15,16]. In addition, these bioactive compounds may influence the expression of genes involved in these processes, notably inflammation and oxidative stress [17]. As such, there is growing evidence that personalized and natural therapeutic approaches could be a promising alternative to prevent cancer progression [18].

Some studies have already explored the effect of compounds derived from olive oil in melanoma. D’Angelo et al. found that hydroxytyrosol, a phenolic compound, has a protective effect in UVA-irradiated melanoma cells, decreasing oxidative damage at lower concentrations (100–400 µM), while at higher concentrations, it inhibits cell proliferation and activates caspase 3, thereby promoting cell apoptosis [19]. Oleic acid, a monounsaturated fatty acid, is also thought to have a protective role against malignancy, reducing cell invasiveness by inhibiting the secretion of SPARC and cathepsin B in melanoma cell lines [20]. However, another study has recently reported that oleic acid did not influence the proliferative capability and melanogenesis of human melanocytes [21]. Thus far, no study has reported the effect of homovanillyl alcohol, a metabolite of hydroxytyrosol, on the viability or aggressiveness of melanoma cells. However, this compound has also been associated with some health benefits, including lower risk for cardiovascular disease across the elderly population [22] and the ability to decrease oxidative stress by scavenging ROS [23,24]. Still, the lack of information about the underlying mechanisms that might explain the reported beneficial effects of olive oil-derived compounds on melanoma remains. Specifically, the effects of hydroxytyrosol, oleic acid, and homovanillyl alcohol, which belong to distinct chemical classes of olive oil compounds, on melanoma are not fully understood yet, since the few available data are controversial. By exploring different classes of olive oil compounds, with distinct features and mechanisms, it might be possible to identify the most promising group of compounds associated with the beneficial effects of olive oil.

In this context, we analyzed the potential of three olive oil compounds: oleic acid, homovanillyl alcohol, and hydroxytyrosol as cytotoxic agents and their implications for the metabolic reprogramming of melanoma cell models.

## 2. Results

### 2.1. Impact of Oleic Acid, Homovanillyl Alcohol, and Hydroxytyrosol on A375 and MNT1 Melanoma Cell Viability

**Figure 1:**
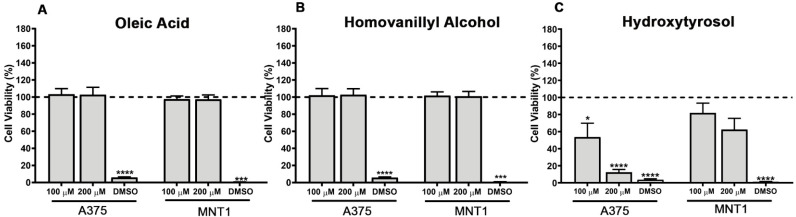
Effects of (**A**) oleic acid, (**B**) homovanillyl alcohol, and (**C**) hydroxytyrosol treatment at concentrations of 100 µM and 200 µM on the metabolic viability of A375 and MNT1 cells, 48 h post incubation. Cell viability of untreated control cells is represented by the dashed line at 100%. Cells treated with 5% dimethyl sulfoxide (DMSO) were used as a positive control of cell viability. Results are representative of at least three independent experiments, performed in triplicate. Data obtained are shown as mean ± standard error of the mean (SEM). Student’s *t*-test was used to compare each experimental condition with the respective untreated control, * *p* < 0.05, *** *p* < 0.001, **** *p* < 0.0001.

The effects of oleic acid, homovanillyl alcohol, and hydroxytyrosol on CM were assessed using viability assays performed 48 h post treatment of two BRAF mutated melanoma cell lines (A375 and MNT1). The concentrations tested were selected based on the literature available about the effects of these compounds on different types of cancer, as well as on the reported concentrations of oleic acid in plasma [19,20,25,26,27,28,29,30]. The treatment with oleic acid and homovanillyl alcohol did not affect the viability of either melanoma cell line in a statistically significant manner (Figure 1A,B). However, the treatment with 100 µM and 200 µM of hydroxytyrosol significantly reduced the viability (*p* = 0.0249; *p* < 0.0001) of A375 cells to approximately 50% and 15% compared to control cells, respectively (Figure 1C). Interestingly, this phenolic compound did not have the same impact on MNT1 cells, but there was a trend for viability reduction, mainly when these cells were treated with a higher concentration of hydroxytyrosol (200 µM).

### 2.2. Metabolic Gene Expression in A375 and MNT1 Melanoma Cells

**Figure 2:**
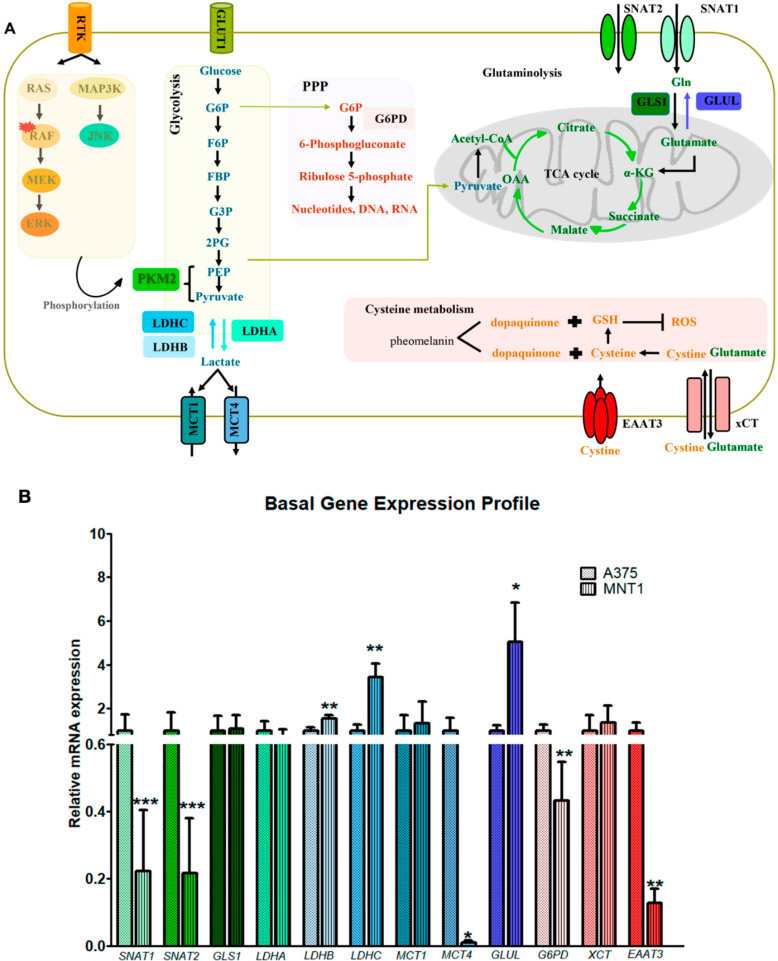
Metabolic characterization of A375 and MNT1 melanoma cells. (**A**) Schematic representation of the main metabolic pathways activated in melanoma cells and their association with MAPK pathways (c-Jun N-terminal kinase (JNK) and extracellular signal-regulated kinase (ERK) activation). Glycolysis, pentose phosphate pathway (PPP), TCA cycle, glutaminolysis, and cysteine metabolism are the main metabolic pathways represented. (**B**) Expression analysis of several genes involved in the metabolic pathways previously mentioned in A375 and MNT1 cells by RT qPCR. Error bars represent the standard deviation of mean values obtained from triplicate experiments. Student’s *t*-test was used to compare two independent groups * *p* < 0.05; ** *p* < 0.01; and *** *p* < 0.001.

MNT1 cells seem to be more resistant to the cytotoxic effect exerted by hydroxytyrosol than A375 cells. In this context, we hypothesized that these two cell models have different metabolic profiles, and we evaluated the expression of genes involved in glutamine and lactate transport and metabolism, pentose phosphate pathway and cysteine transport, hereinafter referred to as metabolic gene expression (Figure 2A). Molecular and metabolic pathways could impact melanoma survival. Rat sarcoma (RAS)/rapidly accelerated fibrosarcoma, (RAF)/mitogen-activated protein kinase (MEK)/extracellular signal-regulated kinase (ERK), and mitogen-activated protein kinase kinase kinase (MAP3K)/c-Jun N-terminal kinase (JNK) pathways mediate pyruvate kinase M2 (PKM2) phosphorylation, ultimately promoting glycolysis. In glycolysis, glucose is converted into pyruvate after several enzymatic reactions involving the following substrates: glucose 6 phosphate (G6P), fructose-6-phosphate (F6P), fructose-1,6-biphosphate (FBP), glyceraldeyde-3-phosphate (G3P), 2-phosphoglycerate (2PG), and phosphoenolpyruvate (PEP). Pyruvate is then converted into lactate by lactate dehydrogenase A (LDHA), and the opposite reaction is mediated by lactate dehydrogenase B and C (LDHB and LDHC). Monocarboxylate transporter 1 and 4 (MCT1 and MCT4) are responsible for lactate import and export from the intracellular space, respectively. In the pentose phosphate pathway (PPP), glucose-6-phosphate dehydrogenase (G6PD) converts glucose-6-phosphate into 6-phosphogluconate. Glutamine is transported to the intracellular medium mainly through Sodium-coupled neutral amino acid transporter 1 and 2 (SNAT1 and SNAT2). Thereafter, glutamine can be converted into glutamate by glutaminase 1 (GLS1), which will supply the TCA cycle by promoting α ketoglutarate (α-KG) production. Contrarily, glutamine synthetase (GLUL) promotes glutamine synthesis via glutamate. To prevent the oxidative stress induced by ROS and maintaining redox balance, melanoma cells possess the ability to induce antioxidant adaptive mechanisms, namely through glutathione (GSH) biosynthesis. Cystine uptake by the transporter cystine glutamate transporter (xCT) and excitatory amino acid transporter 3 (EAAT3) is of the utmost importance to ensure cell detoxification mechanisms (Figure 2A).

This gene expression analysis showed the downregulation of genes encoding transporters involved in the uptake of neutral α-amino-acids, mostly glutamine, such as *SNAT1* and *SNAT2* in MNT1 cells (*p* = 0.0002; *p* = 0.0014) (Figure 2B). Considering that these transporters are mainly responsible for glutamine uptake, these results suggest that A375 cells are more dependent on the exogenous supply of glutamine and other neutral amino-acids as alternative energy sources compared to MNT1. Interestingly, *LDHB* and *LDHC*, but not *LDHA* were upregulated in MNT1 cells (*p* = 0.0025; *p* = 0.0018). *LDHB* and *LDHC* genes encode enzymes that convert l-lactate into pyruvate when oxygen is abundant, while *LDHA* encodes an isoform responsible for the opposite reaction—conversion of pyruvate into l-lactate, in anaerobic conditions, as previously represented in Figure 2A. According to these data, MNT1 cells seem to be more dependent on oxidative metabolism compared to A375 cells. In addition, *MCT4*, which encodes a transporter that facilitates lactate efflux, was downregulated in MNT1 cells (*p* = 0.0128). The downregulation of this transporter could corroborate with the idea that large amounts of lactate remain in the intracellular medium, being directed to the TCA cycle. No differences were found in *MCT1* expression, seeming to indicate that lactate import is not affected. The *GLUL* gene encodes an enzyme, glutamine synthetase, crucial for glutamate conversion into glutamine and it was found upregulated in MNT1 compared to A375 cells (*p* = 0.0256), which indicates that MNT1 cells are less dependent on the exogenous supply of glutamine. *G6PD* was also downregulated in MNT1 cells, probably suggesting that glucose-6-phosphate is being directed for glycolysis and not to PPP (*p* = 0.0081). EAAT3 was also downregulated in MNT1 cells (*p* = 0.0031), although no differences were observed in *xCT* expression.

### 2.3. Effects of Oleic Acid, Homovanillyl Alcohol, and Hydroxytyrosol Induced Metabolic Gene Expression Changes on A375 and MNT1 Melanoma Cells

Further, we assessed the metabolic changes promoted by oleic acid, homovanillyl alcohol, and hydroxytyrosol on both melanoma cell models. To clarify the distinct effect of hydroxytyrosol on both melanoma cell lines, the activation of several metabolic pathways was assessed to detect whether the differences in viability were dependent on the activation of specific metabolic pathways in each cell line. For that purpose, A375 and MNT1 melanoma cells were treated with the same concentrations of hydroxytyrosol used in the viability assays. Despite oleic acid and homovanillyl alcohol showing no effect on melanoma cell viability, we also investigated if the absence of a cytotoxic effect was due to the activation of metabolic pathways.

The treatment with oleic acid (100 µM) induced *LDHA*, *LDHB*, and *LDHC* downregulation in MNT1 cells (*p* = 0.043; *p* = 0.039; *p* = 0.042, respectively) (Table 1; Figure S1). Considering that all these genes belong to the same family, oleic acid seems to affect the conversion of pyruvate into lactate, as well as the opposite reaction. Consequently, the energy produced through glycolysis is significantly reduced after oleic acid treatment. According to the previous results (Figure 2B), MNT1 cells seem to be more dependent on an oxidative metabolism and less dependent on the exogenous supply of glutamine. Thus, it is likely that deficiencies in the glycolytic rate might not be determinant in causing cytotoxic effects. Contrarily to MNT1 cells, oleic acid treatment did not significantly alter the expression of any of the analyzed genes in A375 cells (Table 1; Figure S1).

On the other hand, homovanillyl alcohol at a concentration of 100 µM increased *xCT* expression in A375 cells (*p* = 0.047) (Table 1; Figure S1), which could be related to the efficient mechanisms of cell defense in reverting the oxidative stress as an indirect consequence of the treatment with this compound. The same was not observed in MNT1 cells.

Furthermore, the treatment with 200 µM hydroxytyrosol seemed to significantly increase *SNAT1* expression (*p* = 0.017) and a trend towards an increase of *SNAT2* and *GLS1* expression was also verified in A375 cells (Table 1; Figure S1). In fact, 200 µM hydroxytyrosol treatment reduced A375 cell viability, which is consistent with the increased uptake of neutral amino acids as alternative energy sources to increase the energetic yield as a mechanism to overcome the harmful effects caused by this compound. Hence, the stress condition created by the treatment with this compound may increase the need for new energy sources to resist the toxicity caused by hydroxytyrosol treatment. Moreover, 200 µM hydroxytyrosol also induced *xCT* upregulation and *EAAT3* downregulation (*p* = 0.009; *p* = 0.008, respectively). The increased *xCT* expression can have a pro-survival function under stress conditions, by promoting higher levels of cystine uptake and glutathione biosynthesis, as an attempt by A375 cells to protect themselves from the oxidative stress generated by hydroxytyrosol. Indeed, this result is in accordance with the cytotoxic effect observed in A375 cells after treatment with 200 µM hydroxytyrosol, showing that the cells are activating detoxification mechanisms to survive. Lastly, the treatment with 200 µM hydroxytyrosol significantly decreased *LDHA* and *LDHC* expression in MNT1 cells (*p* = 0.037; *p* = 0.012; *p* = 0.042) (Table 1; Figure S1) preventing the conversion of pyruvate into lactate and the opposite reaction, ultimately inhibiting glycolysis.

### 2.4. Effects of Oleic Acid, Homovanillyl Alcohol, and Hydroxytyrosol on the Activation of ERK and JNK Molecular Pathways in A375 and MNT1 Melanoma Cells

The previous results suggest that the glycolytic process of the selected melanoma cell models is significantly affected mainly by oleic acid and hydroxytyrosol treatments (Table 1). From the three types of MAPK pathways, ERK and JNK have been indicated as the main regulators of energy harvest through glycolysis in melanoma (Figure 2A) [31]. Indeed, these signaling cascades control distinct cellular functions such as cell proliferation, survival, differentiation, and senescence [32]. Particularly, in melanoma cells containing a BRAF mutation, it is thought that glycolysis is enhanced and oxidative metabolism suppressed, promoting resistance to energy stress through ERK activation [33]. To investigate if the differences found in cell viability after hydroxytyrosol treatment and in melanoma metabolism upon treatment with olive oil compounds could be associated with the differential activation of two families of MAPK pathways, ERK and JNK activation was evaluated by Western blot.

**Figure 3:**
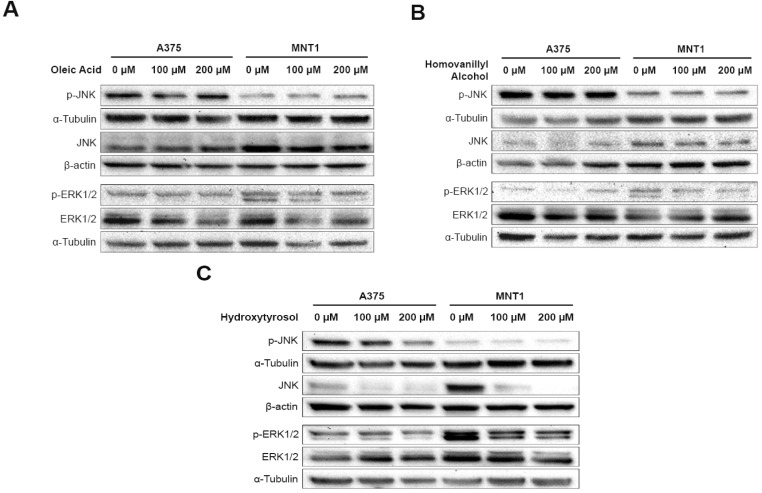
Impact of (**A**) oleic acid, (**B**) homovanillyl alcohol, and (**C**) hydroxytyrosol at concentrations of 100 µM and 200 µM on ERK and JNK pathway activation in A375 and MNT1 cells. Protein expression was detected by Western blot after 48 h of exposure to the three olive oil compounds.

In basal conditions, differences in ERK and JNK pathway activation were observed between A375 and MNT1 cell lines. Higher levels of JNK phosphorylation were detected in A375, and increased ERK activation was verified in MNT1, reflected by its phosphorylation on residues required for the activity of this protein (Figure 3).

In addition, the treatment with 200 µM oleic acid induced a slight increase in JNK activation and a reduction in phosphorylated ERK expression in MNT1 cells (Figure 3A). Since our results suggest that this cell line is more dependent on ERK pathway activation, the treatment with this compound may alter the preferential survival pathway selected by these cells to overcome the microenvironmental changes caused. This trend was not observed in A375 cells. Similarly, a decrease in the levels of phosphorylated ERK was also detected in MNT1 cells after treatment with homovanillyl alcohol (Figure 3B). Overall, oleic acid and homovanillyl alcohol treatments suppressed ERK activation in MNT1 cells.

Finally, hydroxytyrosol treatment reduced the total expression of JNK protein, as well as the levels of phosphorylated ERK, in A375 cells. Similarly, in MNT1 cells, the levels of total JNK expression and phosphorylated ERK were also reduced.

## 3. Discussion

Various compounds present in olives and olive oil have been described as possessing antioxidant, anti-carcinogenic, anti-inflammatory, and neuro-protective properties [15,16]. As such, we sought to analyze the cytotoxic potential of three different olive oil compounds—oleic acid, homovanillyl alcohol (hydroxytyrosol metabolite), and hydroxytyrosol—on melanoma cell models, in order to clarify whether they could represent promising anti-cancer agents to be tested in pre-clinical melanoma models. From the three compounds tested, only hydroxytyrosol had a statistically significant cytotoxic effect on A375 cells, inhibiting their proliferative capability. MNT1 cells seem to be more resistant to the effect of this compound, although there is a tendency to a reduction in viability after treatment. The concentrations tested in this study were selected based on the available literature on the effect of these compounds in other types of cancer and also on the available data about plasma concentrations of oleic acid in a healthy population [28,29,30].

**Figure 4:**
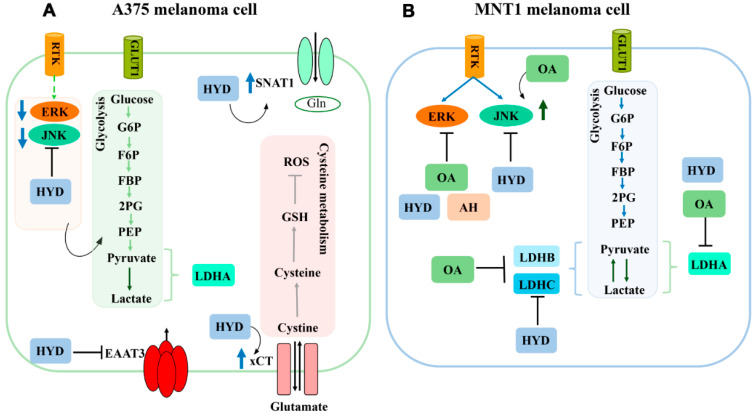
Schematic illustration summarizing the specific effects of oleic acid (OA), homovanillyl alcohol (HA), and hydroxytyrosol (HYD) on key metabolic pathways activated in A375 and MNT1 melanoma cell lines. (**A**) Hydroxytyrosol treatment seems to have a significant effect on A375 cells, reducing total JNK and phosphorylated ERK levels, and consequently decreasing the glycolytic rate via PKM2 inhibition. This compound also increased SNAT1 and xCT expression, probably in order to activate alternative mechanisms of energy production and detoxification in an attempt to resist the cytotoxic stimulus induced by hydroxytyrosol. EAAT3 downregulation could suggest that these cells are less dependent on the transport of l-glutamate, l-aspartate, and d-aspartate. (**B**) In MNT1 cells, hydroxytyrosol also reduced total JNK and phosphorylated ERK levels. In addition, homovanillyl alcohol and oleic acid decreased ERK phosphorylation. The glycolytic rate of this melanoma cell line was also affected by hydroxytyrosol and oleic acid treatments.

Considering the differences induced by hydroxytyrosol treatment in the viability of both melanoma cell models analyzed, we hypothesized that this effect could be mediated by the distinct molecular and metabolic mechanisms preferentially activated in each cell line. We found that both cell models used rely on distinct molecular and metabolic mechanisms, even in the absence of treatment. A375 cells seem to be more dependent on the JNK pathway and MNT1 cells may be more dependent on ERK activation to ensure cell survival and proliferation. In addition, MNT1 cells manifest a predominantly oxidative metabolism compared to A375 cells, as supported by *LDHB* and *LDHC* upregulation, and *MCT4* downregulation. The differential effect caused by hydroxytyrosol could be mediated by the distinct molecular and metabolic pathways predominantly active in each cell line, which could lead to different responses after treatment with this compound. Indeed, hydroxytyrosol treatment led to *SNAT1* and *xCT* upregulation, and to *EAAT3* downregulation in A375 cells, while inducing only *LDHA* and *LDHC* downregulation in MNT1 cells (Figure 4). However, it also resulted in a decrease in total JNK as well as in ERK phosphorylation in both melanoma cell lines. By contrast, oleic acid and homovanillyl alcohol do not seem to affect the viability of either melanoma cell line. However, that does not exclude the possibility of these compounds being able to activate compensatory mechanisms to overcome the stress caused intracellularly, resisting cell death. Oleic acid treatment seems to enhance JNK phosphorylation and reduce ERK pathway activation in MNT1 cells, while also inhibiting *LDHA*, *LDHB* and *LDHC* expression and, consequently, the energy produced through glycolysis (Figure 4). In addition, the treatment with homovanillyl alcohol had a suppressive effect on phosphorylated ERK activation in MNT1 cells. Regarding A375 cells, homovanillyl alcohol increased *xCT* expression at a lower concentration.

Presently there is evidence showing that hydroxytyrosol is an anticancer agent in various human cancers including colorectal, breast, thyroid, digestive, lung, brain, blood, and cervical cancers (reviewed in [34]). In fact, several studies have reported the anti-tumoral effect of hydroxytyrosol on different types of cancer, and the distinct anti-proliferative and pro-apoptotic mechanisms triggered in each cancer cell type (reviewed in [35]). In human breast cancer MCF-7 cells, hydroxytyrosol treatment reduced cell viability and proliferation, and promoted cell apoptosis [36]. In addition, in LNCaP prostate cancer cells, hydroxytyrosol inhibited cell proliferation in a concentration dependent manner (IC50 = 176 µM 48 h post treatment) through the suppression of Akt/STAT3 phosphorylation and by the cytoplasmic retention of NF-κB [37]. Nevertheless, hydroxytyrosol did not affect the viability of normal prostate epithelial cells, indicating a targeted effect against cancer cells [37].

In cutaneous melanoma cell lines, a dual role of hydroxytyrosol was previously reported when used in different concentrations (0–1000 µM) [19]. In lower concentrations (100–400 µM), this phenolic compound was photoprotective against ultraviolet radiation A, although in higher concentrations (600–1000 µM) it inhibited cell proliferation and activated caspase 3, consequently promoting cell apoptosis [19]. According to this study, hydroxytyrosol has a dual role in melanoma, based on the concentration administered, which is of utmost relevance since these compounds are present in cosmetics and functional foods. In our study, we did not address the range of concentrations tested by the previous authors. However, similarly to the aforementioned study, our results might corroborate a cytotoxic effect of this compound according to the mechanisms predominantly active in melanoma cells.

The distinct behavior of A375 and MNT1 cells after hydroxytyrosol treatment is consistent with the hypothesis that both melanoma cell lines rely on different predominant molecular and metabolic mechanisms to survive, which is also expected since A375 and MNT1 correspond to a primary [38] and metastatic [39] melanoma cell line, respectively. Previous studies postulate that metastatic melanoma cells differ from non-metastatic melanoma and normal melanocytes by preferentially showing higher levels of oxidative metabolism, fatty acid oxidation, and glutaminolysis [40,41,42]. Perhaps by increasing the oxidation of specific substrates, metastatic melanoma cells are able to acquire the spare energy they need to migrate and invade other tissues [41]. Indeed, MNT1 cells seem to maintain lactate in the intracellular environment, reducing its export by MCT4, certainly to be converted into pyruvate by LDHB and LDHC [43,44,45], which will then supply the TCA cycle and enhance the oxidative phosphorylation cascade [46,47]. These results are in accordance with the idea that a subset of melanomas rely extensively on oxidative phosphorylation to meet their bioenergetic needs, which could condition therapy response. Since most metastatic melanomas rely on a more oxidative metabolism, the study of targets involved in these metabolic pathways should be explored.

In addition, MNT1 cells seem to be less dependent on the exogenous supply of glutamine and are able to obtain this amino acid through glutamate conversion [48], which could explain the downregulation of *SNAT1* and *SNAT2*, and *GLUL* upregulation. These metastatic cells also seem to direct glucose-6-phosphate to glycolysis instead of being used in the pentose phosphate pathway, which was previously described as an indicator of reduced melanoma aggressiveness [49]. Based on *EAAT3* downregulation, MNT1 are also less dependent on the transport of l-glutamate, l-aspartate, and d-aspartate.

The decrease verified in total JNK as well as in ERK phosphorylation after hydroxytyrosol treatment in A375 and MNT1 cells could possibly have a negative impact on the glycolytic rate, since MAPK pathways are known to increase glycolytic activity though M2 pyruvate kinase (PKM2) activation [32,33,34]. In a condition of nutrient scarcity, melanoma cells try to acquire the available energy sources to maintain cell homeostasis and survival [50]. Consistently, A375 cells also take up glutamine more efficiently and activate detoxification pathways dependent on the xCT transporter, as was previously described for endometrial cancer [51], in an attempt to survive the deleterious effects of hydroxytyrosol treatment [52]. *EAAT3* downregulation could be related to the difficulty of these cells in performing the antiport of cystine and glutamate under stress conditions. On the other hand, in MNT1 cells, this compound increased the levels of pyruvate being directed to the TCA cycle through *LDHA* downregulation. Possibly, the fact that MNT1 cells rely more on an oxidative metabolism might give them an advantage regarding hydroxytyrosol treatment, since *LDHA* downregulation will favor this metabolism, diminishing its cytotoxic effects.

Concerning oleic acid, a study reported that this compound did not influence the proliferative capability and melanogenesis of human melanocytes [21]. Furthermore, a previous study demonstrated that oleic acid was involved in protection against melanoma malignancy, modifying tumor microenvironment and reducing melanoma cell invasiveness [20]. Despite the reduced sampling used (*n* = 51), serum oleic acid was not associated with the risk of CM development [53]. In contrast to what was verified in melanoma, oleic acid was associated with cancer growth and metastasis through the activation of the ERK1/2 pathway in cervical cancer [54]. Perhaps the absence of an effect on melanoma cell viability of oleic acid treatment is due to compensatory and detoxifying mechanisms that are activated. In particular, different survival and metabolic pathways are selected and activated after the addition of this compound to MNT1 cells, as an attempt to overcome the microenvironmental alterations caused. These cells become more dependent on other energy sources such as glutamine to maintain the TCA cycle after oleic acid treatment. Accordingly, a previous study demonstrated that oleic acid can promote an adaptive response and enhance cell tolerance through its increased cellular antioxidative capacity via lipid peroxidation, which ultimately protects cells against oxidative stress-related injury [55]. In addition, it was already reported that the JNK pathway has a suppressive effect on ERK pathway activation, as an attempt to regulate cell apoptosis [56], which is coherent with our observation that oleic acid increases JNK activation while inhibiting ERK phosphorylation. The constant crosstalk between survival and proliferation pathways could be an alternative adaptation that allows resistance to the consequences of cellular stress.

Regarding homovanillyl alcohol, to our knowledge this is the first study exploring the impact of this hydroxytyrosol metabolite on the viability of two different melanoma cell lines. Based on the available literature, the impact of hydroxytyrosol in the context of cancer has been much more explored than the role that oleic acid and homovanillyl alcohol play under pathological conditions. However, no differences were verified in the viability of melanoma cells after homovanillyl alcohol treatment, perhaps meaning that other alternative survival and detoxification mechanisms dependent on xCT transporters are being activated. Under stress conditions, cells use different energy sources to resist and adapt to microenvironmental changes.

## 4. Materials and Methods

### 4.1. Cell Culture

Human melanoma cell lines A375 (malignant, ATCC CRL-1619) and MNT1 (metastatic, ATCC CRL-3450), both BRAF *^V600E^* mutated. A375 cells were cultured in Dulbecco’s modified Eagle’s medium (DMEM) without sodium pyruvate, supplemented with 10% fetal bovine serum (FBS), and 1% penicillin/streptomycin (all from Gibco; Thermo Fisher Scientific, Waltham, MA, USA). MNT1 cells were cultured in DMEM high glucose, supplemented with 10% FBS, 1% penicillin/streptomycin, 1% l-glutamine, and 1% non-essential amino acids (all from Gibco; Thermo Fisher Scientific, Waltham, MA, USA). Both cell lines were grown in a humidified incubator at 37 °C, 5% CO_2_ atmosphere.

### 4.2. Compounds

The exponentially growing melanoma cell lines were treated with 100 µM and 200 µM of three olive oil compounds, namely oleic acid (O1008, Sigma-Aldrich, Merck KGaA, Darmstadt, Germany), homovanillyl alcohol (148830, Sigma-Aldrich, Merck KGaA, Darmstadt, Germany), and hydroxytyrosol (4999 S, Extrasynthese, Genay, France). The concentrations used to treat A375 and MNT1 cells were selected based on the literature available about the effects of these compounds on different types of cancer. In addition, articles describing the concentrations of oleic acid in the plasma of healthy individuals were also considered to choose the concentrations to be used in this study [28,29,30]. Oleic acid was detected in a range of concentrations between 0.03 and 3.2 mmol/L (30 µM and 3200 µM) in plasma of a population of healthy young Canadian adults (*n* = 826) [30]. Stock solutions of 100 mM were prepared in MilliQ water for hydroxytyrosol, homovanillyl alcohol, and oleic acid.

### 4.3. Viability Assays

Cell counting kit-8 (CCK8) (Dojindo, Munich, Germany) was used to analyze cell viability 48 h post treatment with homovanillyl alcohol, oleic acid, and hydroxytyrosol. Cells were seeded in 24-well plates at a density of 1 × 10^5^ cells/well. Cells treated with 5% dimethyl sulfoxide (DMSO) were used as a positive control of cell death. After 48 h, CCK8 solution was added to the wells in a dilution of 1:10 and cells were incubated for 30 min. Four independent assays were performed, and absorption was read at 450 nm using a microplate reader (BioRad, Hercules, CA, USA).

### 4.4. Quantitative Real Time Polymerase Chain Reaction (qRT-PCR)

Cells were seeded in 6-well plates at a density of 2.5 × 10^5^ cells/well and after 24 h treated with 100 µM and 200 µM of three olive oil compounds. RNA was extracted from A375 and MNT1 cells 48 h post treatment with olive oil compounds using NZY Total RNA Isolation kit, following manufacturer instructions (Nzytech, Portugal). RNA quantification was assessed using a NanoDrop^®^ 2000 Spectrophotometer (NanoDrop Technologies, Inc., Wilmington, DE, USA) and cDNA synthesis was performed using 1 µg of RNA, reversely transcribed by SuperScript III Reverse Transcriptase (RT) (Invitrogen, Thermo Fisher Scientific, Waltham, MA, USA) following manufacturer’s recommendations for a final reaction volume of 20 µL.

qRT-PCR reactions were carried out using SYBR Green master mix (Invitrogen, Thermo Fisher Scientific, Waltham, MA, USA) during 40 cycles in Lightcycler® 480 System instrument (Roche, Basel, Switzerland). The amplification program consisted of a holding stage (95 °C, 10 min), followed by amplification cycles (95 °C, 15 s and 60 °C, 1 min). The primers used for the expression analysis of sodium-coupled neutral amino acid transporter 1 and 2 (*SNAT1* and *SNAT2*), glutaminase 1 (*GLS1*), glutamine synthetase (*GLUL*), lactate dehydrogenase A, B, and C (*LDHA*, *LDHB* and *LDHC*), monocarboxylate transporter 1 and 4 (*MCT1* and *MCT4*), glucose-.6-phosphate dehydrogenase (*G6PD*), cystine-glutamate transporter (*xCT*) and excitatory amino acid transporter 3 (*EAAT3*) are listed in Table 2. Hypoxanthine-guanine phosphoribosyltransferase (*HPRT1*) and TATA-box binding protein (*TBP*) were selected as housekeeping genes (Table 1). Gene expression was evaluated by comparative CT method (ΔΔCT) [57] and each gene was normalized to the endogenous human reference housekeeping *TBP* and *HPRT1* genes. Experiments were performed in biological and practical triplicates.

### 4.5. Western Blot

A375 and MNT1 cells were seeded in T25 flasks at a density of 6.5 × 10^5^ cells/flask and after 24 h these cells were treated with 100 µM and 200 µM of oleic acid, homovanillyl alcohol, and hydroxytyrosol. Cells collected 48 h post treatment with olive oil compounds were washed with phosphatase buffered saline and lysed for 1 h using ice-cold RIPA lysis buffer (Gibco, Thermo Fisher Scientific, Waltham, MA, USA) supplemented with a protease and phosphatase inhibitor cocktail (Sigma-Aldrich, Merck KGaA, Darmstadt, Germany). Protein concentration was determined using Pierce BCA protein assay kit (Thermo Fisher Scientific, Waltham, MA, USA) according to the manufacturer’s instructions. Protein extracts (40 μg) were separated using 10% and 12% SDS-polyacrylamide gels and transferred onto PVDF membranes (BioRad, Hercules, CA, USA) with the Trans-Blot^®^ Turbo TM Blotting system (BioRad, Hercules, CA, USA). Immunostaining was achieved using the following primary antibodies: anti-phospho-JNK (Thr183/Tyr185, Thr221/Tyr223) (1:500 dilution; 72 h incubation; 07-175, Merck KGaA, Darmstadt, Germany), anti-JNK/SAPK1 (1:165 dilution; overnight incubation; 06-748, Merck, Darmstadt, Germany), anti-phospho-ERK1/2 (pT202/pY204), clone AW39 (1:125 dilution; 72 h incubation; 612358, BD Biosciences, San Jose, CA, USA), anti-p44/42 MAPK (ERK1/2) (1:500 dilution; 72 h incubation; 168-10069, Raybiotech, Norcross, GA, USA), anti-α-tubulin, clone B-5-1-2 (1:4000 dilution; 1 h incubation; T5168, Merck KGaA, Darmstadt, Germany), and anti-β-actin, clone AC-15 (1:5000 dilution; 1 h incubation; A5441, Merck KGaA, Darmstadt, Germany). Primary antibodies were detected using horseradish peroxidase-conjugated secondary goat anti-rabbit and anti-mouse antibodies (1:5000 dilution; 1 h incubation; 31,460 and 31,430, ThermoFisher Scientific, Waltham, MA, USA), followed by enhanced chemiluminescence detection using Clarity Max Western ECL Substrate (BioRad, Hercules, CA, USA). Membranes were imaged by ChemiDoc XRS+ (BioRad, Hercules, CA, USA).

### 4.6. Statistical Analysis

The differences in the mean viability and gene expression of control cells and cells treated with oleic acid, homovanillyl alcohol, and hydroxytyrosol were assessed by employing Student’s *t*-tests. Sample data were represented as the mean ± standard deviation or the mean ± SEM for the metabolic viability results. All tests were two-sided, and we considered a significance level of 5%. Statistical analysis and graphic representation were performed using GraphPad Prism (version 5).

## 5. Conclusions

This study demonstrates that melanoma cells have a distinct response to natural olive oil compounds, which could be explained by a considerable metabolic and molecular heterogeneity. Here, we showed that oleic acid and homovanillyl alcohol are likely not a reliable therapeutic strategy in melanoma by themselves, because these compounds may facilitate the activation of compensatory mechanisms which increase cell detoxification and/or the dependence on an oxidative metabolism, essential for melanoma cells to resist to stress conditions. Hydroxytyrosol was the only compound to have a significant impact in melanoma cell viability, but this effect was only observed for the primary melanoma cell line. Future studies should be performed using other melanoma cell lines (primary and metastatic), in order to assess whether the impact of these three olive oil compounds is consistently influenced by the specific metabolic and molecular mechanisms predominantly activated in each cell line. Nevertheless, the present study corroborates the idea that hydroxytyrosol could represent a promising compound to be tested in pre-clinical melanoma models, mainly in melanoma tumors with a predominant glycolytic metabolism and preference for JNK pathway activation. Indeed, therapies focused on specific metabolic and molecular features of each melanoma subset could be more efficient since they will rely on distinct targets according to tumor characterization.

## Figures and Tables

**Table 1:** Effects of oleic acid, homovanillyl alcohol, and hydroxytyrosol treatment on metabolic gene expression in A375 and MNT1 cells after 48 h of exposure by RT-qPCR analysis.

	A375 Melanoma Cells						MNT1 Melanoma Cells					
	Oleic Acid		Homovanillyl Alcohol		Hydroxytyrosol		Oleic Acid		Homovanillyl Alcohol		Hydroxytyrosol	
Gene	100 µM	200 µM	100 µM	200 µM	100 µM	200 µM	100 µM	200 µM	100 µM	200 µM	100 µM	200 µM
*SNAT1*	nss	nss	nss	nss	nss	*p* = 0.017	Nss	nss	Nss	nss	nss	nss
*SNAT2*	nss	nss	nss	nss	nss	nss	Nss	nss	Nss	nss	nss	nss
*GLS1*	nss	nss	nss	nss	nss	nss	Nss	nss	Nss	nss	nss	nss
*LDHA*	nss	nss	nss	nss	nss	nss	*p* = 0.043	nss	Nss	nss	*p* = 0.037	*p* = 0.012
*LDHB*	nss	nss	nss	nss	nss	nss	*p* = 0.039	nss	nss	nss	nss	nss
*LDHC*	nss	nss	nss	nss	nss	nss	*p* = 0.042	nss	nss	nss	nss	*p* = 0.042
*MCT1*	nss	nss	nss	nss	nss	nss	Nss	nss	nss	nss	nss	nss
*MCT4*	nss	nss	nss	nss	nss	nss	Nss	nss	nss	nss	nss	nss
*GLUL*	nss	nss	nss	nss	nss	nss	Nss	nss	nss	nss	nss	nss
*G6PD*	nss	nss	nss	nss	nss	nss	Nss	nss	nss	nss	nss	nss
*xCT*	nss	nss	*p* = 0.047	nss	nss	*p* = 0.009	Nss	nss	nss	nss	nss	nss
*EAAT3*	nss	nss	nss	nss	nss	*p* = 0.008	Nss	nss	nss	nss	nss	nss

nss—Not statistically significant. Green—genes upregulated. Red—genes downregulated.

**Table 2:** Primer sequences used for qRT-PCR.

Gene	Primer Forward	Primer Reverse
*SNAT1*	CATTCTATGACAACGTGCAGTCC	CAGCAACAATGACAGCCAGC
*SNAT2*	CTGAGCAATGCGATTGTGGG	CTCCTTCATTGGCAGTCTTC
*GLS1*	CTTCTACTTCCAGCTGTGCTC	CACCAGTAATTGGGCAGAAACC
*GLUL*	GAATGGTCTGAAGTACATCGAGG	GTTAGACGTCGGGCATTGTC
*LDHA*	CTTGCTCTTGTTGATGTCATC	CAGCCGTGATAATGACCAGC
*LDHB*	GAGCCTTCTCTCTCCTGTG	CTGATAGCACACGCCATACC
*LDHC*	GGATCTTCAGCATGGCAGTC	CTATTCTGGAGTTTGCAGATA
*MCT1*	GCTGGGCAGTGGTAATTGGA	CAGTAATTGATTTGGGAAATGCAT
*MCT4*	CACAAGTTCTCCAGTGC	CGCATCCAGGAGTTTGC
*G6PD*	GGCAACAGATACAAGAACGTGAAG	GCAGAAGACGTCCAGGATGAG
*XCT*	GGTCCTGTCACTATTTGGAGC	GAGGAGTTCCACCCAGACTC
*EAAT3*	GTATCACGGCCACATCTGCC	GCAATGATCAGGGTGACATCC
*TBP*	GAGCTGTGATGTGAAGTTTCC	TCTGGGTTTGATCATTCTGTAG
*HPRT1*	TGAGGATTTGGAAAGGGTGT	GAGCACACAGAGGGCTACAA

## Data Availability

The data presented in this study are available on request from the corresponding author.

## Supplementary Materials

The following are available online, Figure S1—Effect of oleic acid, homovanillyl alcohol, and hydroxytyrosol (100 µM and 200 µM) treatment on *SNAT1*, *SNAT2*, *GLS1*, *LDHA*, *LDHB*, *LDHC*, *MCT1*, *MCT4*, *GLUL*, *G6PD*, *EAAT3*, and *xCT* mRNA expression in A375 and MNT1 cells after 48 h of exposure, by RT-qPCR analysis. Error bars represent the standard deviation of mean values obtained from triplicate experiments. Student’s *t*-test was used to compare each experimental condition with the respective untreated control * *p* < 0.05; ** *p* < 0.01.

## Sample Availability

Samples used during the current study are available from the authors on reasonable request.
