# Supplementary figures and images for: The Impact of Olive Oil Compounds on the Metabolic Reprogramming of Cutaneous Melanoma Cell Models
Source: Molecules. 2021 Jan 8;26(2):289. doi: 10.3390/molecules26020289 (PMC7827395; doi:10.3390/molecules26020289)

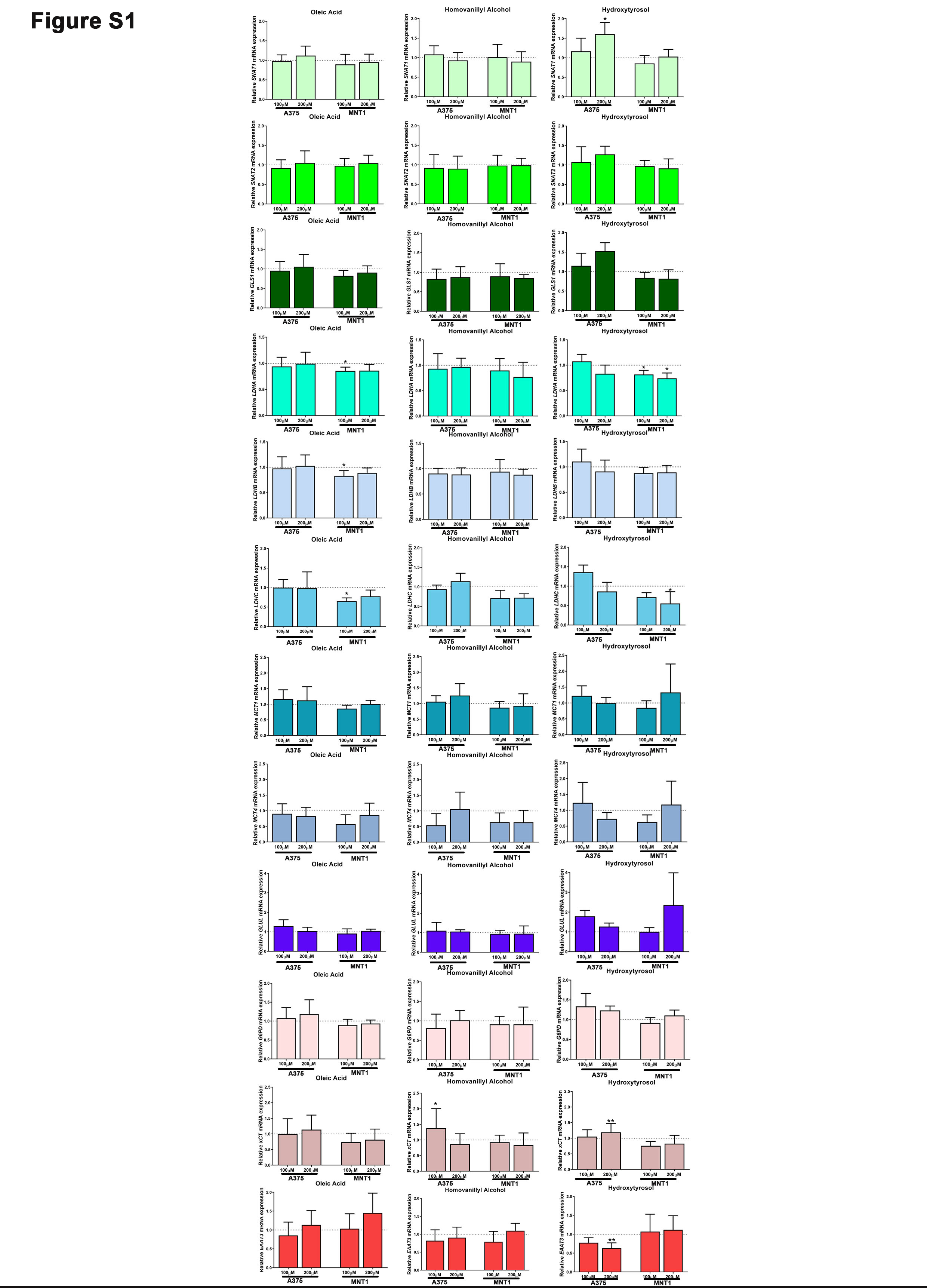

Supplement: Supplementary file 1 [file molecules-26-00289-s001.zip › molecules-1003200-supplementary.tif]
